# Development and Characterization of Thermomechanically Treated and Untreated Banana Rachis Fiber/PLA Composites for Material Extrusion Additive Manufacturing

**DOI:** 10.3390/polym18091144

**Published:** 2026-05-06

**Authors:** Elena Monzón, Pablo Bordón, Quim Tarrés, Mario Monzón, Rubén Paz

**Affiliations:** 1 Integrated and Advanced Manufacturing Research Group, University of Las Palmas de Gran Canaria, 35017 Las Palmas, Spain; pablo.bordon@ulpgc.es (P.B.); ruben.paz@ulpgc.es (R.P.); 2 LEPAMAP-PRODIS Research Group, University of Girona, C/Maria Aurèlia Capmany 61, 17003 Girona, Spain; joaquimagusti.tarres@udg.edu

**Keywords:** additive manufacturing, material extrusion, banana rachis fiber, natural fiber, polylactic acid, natural fiber composites, sustainable composites, biodegradable composites

## Abstract

In this study, a biodegradable composite based on PLA reinforced with banana rachis fiber—derived from agricultural waste and forming the structural core of banana bunches—is developed. The fibers are evaluated with and without thermomechanical processing to enhance the properties of parts produced through material extrusion additive manufacturing (MEX), a technology with a screw feeding system. A preliminary study of the additive manufacturing process is conducted to ensure adequate processability of the matrix during the process. In addition, different composite formulations (0, 5, 10 and 15 wt.% fiber) are analyzed through morphological, thermal (TGA and DSC), rheological, and mechanical characterization, complemented by SEM analysis. This comprehensive characterization revealed that the incorporation of WTP fibers served to reinforce the PLA matrix for the tensile modulus, from 2273.54 ± 123.66 MPa to 2612.51 ± 95.16 MPa with 15% of WTP fiber. A similar trend was observed for the flexural modulus, which increased from 2456 ± 61.16 MPa in the neat PLA to 3189.68 ± 52.24 MPa for the PLA-15% WTP composite. The results demonstrate the feasibility of the process and the production of parts with acceptable quality under appropriate manufacturing conditions.

## 1. Introduction

The development of composite materials is widely pursued to enhance material properties and lighten the weight of the parts at a lower cost. Consequently, these materials are employed in diverse sectors, including automobile, aerospace, packaging and building industries [1]. In contrast to conventional metallic materials and alloys, the use of synthetic fiber composites offers significant advantages in specific strength and durability, leading to their widespread industrial adoption [2]. Several reinforcement materials have been incorporated into polymer matrices, such as carbon, aramid or glass fiber. Among these, glass fiber is the most commonly used due to its favorable mechanical properties and its low cost, compared to other synthetic fibers [3]. However, the extensive industrial use of this type of non-degradable composite has led to significant disposal challenges due to the massive volume of waste. To reduce the environmental impact caused by non-biodegradable materials, the focus has been placed on ecological composites such as plant fibers, allowing different sectors, including agriculture, forestry or industry to take advantage of the residues and providing additional value to natural waste [2]. The diversity of these residues expands the range of sustainable materials that can be used for research or manufacturing purposes.

For example, Badouard et al. developed composites made with flax fibers and three types of polymer matrices: Poly-(lactid) (PLLA), Poly-(butyl-adipate-terephthalate) (PBAT) and Poly-(butylene-succinate) (PBS). The study observed that the matrix selected is relevant to control the mechanical behavior of the composite. In addition, the fiber volume fractions that the polymer can support depend on their initial elongation [4]. Stoof and Pickering, who obtained composites with different materials such as recycled gypsum and pre-consumer recycled polypropylene (PP), utilized harakeke and hemp fiber as reinforcement [5]. They analyzed a new method of measuring the shrinkage of parts produced with additive manufacturing.

Along with the literature, there are plenty of studies that incorporate natural fibers in their composite to promote a circular economy and reduce non-recyclable material. Cabrera et al. analyzed the environmental impact of the production of the recycled high-density polyethylene matrix and banana rachis fiber utilizing a life cycle assessment approach. They concluded that incorporating the fiber in the composites produces a reduction in the environmental impacts of the material when the reinforcement increases. The study shows a decrease of 69% carbon emissions for the composite with 5% of fiber and recycled polymer compared to a virgin matrix [6]. Chaitanya et al. examined the effect of recycling the composite eight times. This compound was made with PLA and 30% treated Sisal fiber through the extrusion process. The results were analyzed through mechanical and thermal properties, showing a decline in tensile and flexural strength by 20.9% and 21.2%, respectively, up to the third recycling. The multiple recyclings showed the degradation of the material, which is one of the causes of the hydrolysis. The researchers concluded not to recycle the composite beyond the third recycling, albeit it can be utilized until the third cycle to manufacture components for non-structural applications for low to medium strength [7].

To improve the adherence between the matrix and the reinforcement and therefore enhance the properties of the composites, it is common to apply treatments to natural fibers. For example, Oliveira Camillo et al. analyzed the properties of a composite made with castor-oil-based polyurethane and coconut coir fibers, with and without hydrothermal treatment. The study concluded that the treatment produces a reduction in the extractive content in the fiber, improving the interfacial adhesion between the matrix and the reinforcement, which serves to elevate the composite’s flexural strength and elastic modulus [8].

A widely studied biomass is banana plant fiber, with the pseudostem, leaves, and rachis; this last component is the main nucleus of the bunch that supports the bunches of bananas once the fruit is harvested from the plant [9]. The pseudostem and leaves are usually left to decompose in banana plantations, whereas the rachises are milled at banana processing facilities. Valorizing these residues promotes a circular economy, allowing the development of new applications for the waste. Products such as threads, ropes, or baskets can be produced. However, compared to the pseudostem, the use of rachis fiber simplifies the collection of the by-product, as it is collected at processing centers along with the fruit clusters, facilitating product management and cutting costs, which makes it an interesting material to be analyzed [9,10].

Diverse procedures such as injection or compression molding have been adopted to explore biocomposite materials, including additive manufacturing (AM). In recent years, AM has been a remarkable technology, enabling the obtaining of products with complex geometries, prototype development, customized products, and the reduction of material waste. Among different types of methods used in AM, material extrusion (MEX, also well known as FDM) is one of the most extensive due to its simplicity and low cost. The manufactured parts are built layer by layer through the deposition of the material, which is fed into the AM equipment in a filament format. To avoid generating the filament, which is an additional process to obtain the initial material for AM, the literature shows the use of pellets instead of filaments. This allows avoiding the strict dimensional control of the filament criteria to reduce the warping effect or avoid the blocking of the equipment feed mechanism. A further constraint of the use of this material format involves the narrow range of thermoplastic polymers presented in the market [11].

To analyze whether there are differences in the mechanical properties between parts manufactured in AM through filament or pellet forms, Liu et al. compared their properties and concluded that there were no differences in the mechanical behavior [12]. Fontana et al. examined the porosity of samples manufactured with PLA in pellet form, determining that an increase in the volume of extruded material correlates with a higher incidence of micropores. Moreover, the study evaluated the energy consumption of the AM machine throughout the sampling process, showing that when the printing speed increases, it leads to lower energy consumption while preserving the material strength [11]. Thanks to the versatility of this technology, different applications can be found when additive manufacturing systems are used, such as Shi et al., who utilized AM to fabricate components for electromagnetic interference shielding for smart electronic devices using a PLA/graphene nanoparticle composite [13].

Biobased materials are commonly used in additive manufacturing, such as Wang et al., who investigated the recyclability of flax and polypropylene composite during several cycles, or Pintos et al., who valorized the olive pit agro-waste, adding it in a polyethylene terephthalate glycol (PETG) matrix, using a large format of additive manufacturing [14,15]. As shown, different studies have investigated the use of additive manufacturing and natural composites. However, to the best of the authors’ knowledge, few studies have analyzed composites with rachis fiber, and none have utilized AM with a composite made with a biobased polymer such as PLA and rachis fiber in a pellet format [9]. Due to the well-known advantages of this technology and the benefits of valorizing rachis fiber, the present research investigates the processability of rachis fiber-reinforced PLA composites via material extrusion (MEX). Initially, printing parameters were calibrated using neat PLA and a 15 wt% untreated fiber composite to evaluate the influence of nozzle diameters (0.8 and 1.0 mm) on flexural properties. To enhance interfacial adhesion and mechanical performance of the composite, thermomechanical processing (TP) was applied to the fibers, and a comprehensive characterization was conducted to compare AM composites with and without thermomechanical processing across different reinforcement loadings (5, 10, and 15 wt%).

## 2. Materials and Methods

### 2.1. Fiber Extraction

The fibers analyzed in this study were extracted from the rachis of the banana plant variety Musa acuminata (Cavendish Group), obtained from plantations located in the north of Gran Canaria (Canary Islands, Spain).

**Figure 1:**
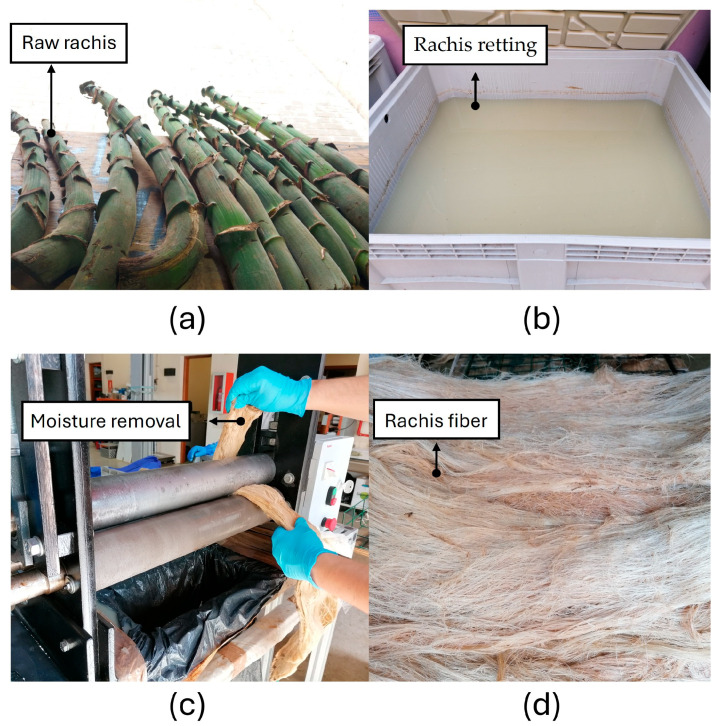
Fiber extraction; (**a**) rachis: the central core of the banana bunch, which serves as the main support structure; (**b**) water retting; (**c**) mechanical compression: this process is employed to remove part of the water absorbed by the fiber; (**d**) sun-drying.

The process to extract the rachis fibers consisted of several stages. First, the external layer of the rachis was manually removed. Then, the rachis underwent water retting for approximately 46 days at room temperature. After retting, the material was manually cleaned with water, followed by mechanical compression through compressive rolls to remove excess moisture. Finally, the fibers were slightly separated using compressed air to facilitate the final sun-drying stage (Figure 1).

### 2.2. Fiber Processing

Different settings were assessed to process both the fiber with and without thermomechanical processing. When TP is carried out, fibers with thermomechanical processing (WTP) should become more individualized, resulting in a larger aspect ratio (length to width), which could improve the adherence between the matrix and the fiber. In the case of fibers without thermomechanical processing (WOTP), in order to incorporate them into the matrix and obtain a suitable size for AM, the fibers were milled at 2100 rpm with a sieve of 1 mm using a Retsch SM 300 mill (Haan, Germany). Subsequently, the fibers passed through a vibratory sieve with a mesh size of 400 µm to standardize particle size.

For the thermomechanical process (WTP compounding), which causes breakage and reduces fiber length, the fibers were previously milled and sieved using the same Retsch SM machine at 1500 rpm with a 20 mm sieve to obtain larger fibers for the TP process.

**Figure 2:**
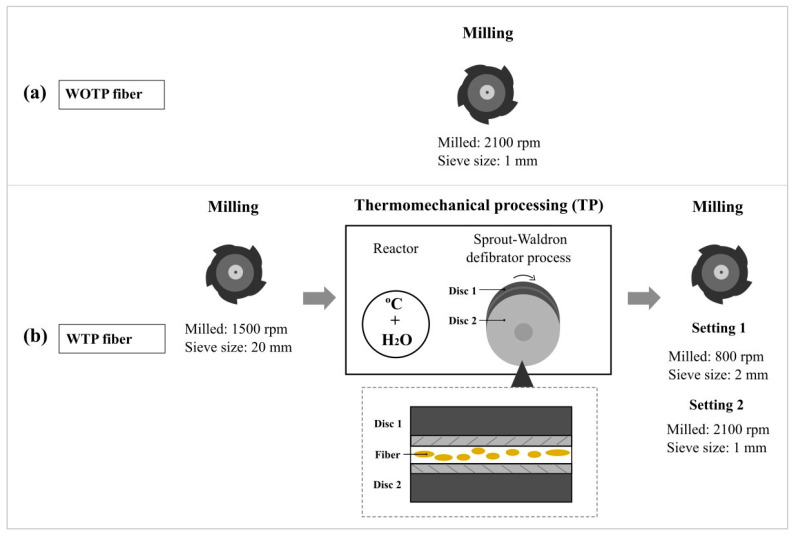
Fiber processing: (**a**) fiber without thermomechanical processing (WOTP): a milling process is performed; (**b**) fiber with thermomechanical processing (WTP): first, the fiber is milled, second, a thermomechanical process is performed, and finally, two different fiber configurations are obtained.

The TP was carried out in two stages. In the first stage, parts of extractives and lignin were removed using a conventional rotary reactor preheated to 80 °C. The fiber was treated in a 6:1 water:fiber ratio at 180 °C for an hour and then washed with water to remove the residual extractives. In the second stage, the fiber underwent a defibrator process to individualize the fiber (Sprout-Waldron, Muncy, PA, USA). This process uses two discs with channels that rotate to separate the fibers. After defibration, the material was air-dried. To prepare the WTP fiber for composite manufacturing, two final cutting settings were applied: one portion was milled and sieved at 800 rpm with a 2 mm sieve to increase fiber size, and the second portion was milled using the same settings as for the WOTP fiber to allow the comparison between the two composites. The fiber processing is summarized in Figure 2.

### 2.3. Composite Manufacturing

Nine composite formulations were developed using Luminy^®^ PLA L105 (TotalEnergies Corbion, Gorinchem, The Netherlands) (Table 1). The formulations combined three fiber contents (5, 10, and 15 wt.%), two fiber treatments (WOTP and WTP), and two milling conditions applied to WTP fibers (800 and 2100 rpm). The components of neat PLA are summarized in Table 2, based on the manufacturer’s technical data sheet.

Compounding was performed using a filament extruder (Thermo Fisher Scientific Inc., Karlsruhe, Germany) with a 2 mm die and the following temperature profile: 175 °C (die zone), 180 °C, 180 °C, 185 °C, 185 °C, 175 °C, 175 °C, and 165 °C. Both the fiber and PLA were dried in an oven at 105 °C for 4 h prior to compounding. Finally, the filament was pelletized using a pellet mill (Thermo Fisher Scientific Inc., Karlsruhe, Germany).

### 2.4. Additive Manufacturing

In this study, additive manufacturing was performed using an Ender 3 AM machine (Shenzhen Creality 3D Technology Co., Ltd., Shenzhen, China). To enable pellet feedstock processing, the machine was modified by adding a V4 pellet extruder (Mahor.xyz, Navarra, Spain). The extruder features a modular configuration with an extrusion screw and stepper motor system optimized for pellet-based printing. The slicer software used was Ultimaker Cura 5.4.0. This study was carried out using the constant AM settings listed in Table 3. The parameters were defined based on conventional settings within the additive manufacturing field, as well as by carrying out preliminary material processing tests.

### 2.5. Preliminary Study of Composite Processing by AM

To address the complexity of pellet-based AM using natural-fiber composites and to determine the optimal process configuration, a preliminary analysis was carried out to evaluate the processability of the 15 wt.% fiber composite, which is the maximum loading achieved in previous tests by the authors. Different parameters were evaluated. First, nozzle diameters of 0.8 and 1.0 mm were selected based on common standards for printing natural-fiber composites. While a 0.6 mm nozzle was initially tested, it resulted in persistent obstruction; consequently, this diameter was discounted for the remainder of the study. The use of pellets in AM requires adjusting the extruder stepper motor to ensure adequate flow of material. Therefore, stepper motor calibration was performed for both nozzles (1 and 0.8 mm) using neat PLA as a reference.

Secondly, to calibrate the motor steps, density measurements were conducted on PLA pellets to calculate the required mass flow. Finally, a preliminary study of the flexural properties was carried out on parts made with neat PLA and with PLA 15–WOTP (composite with 15% fiber without thermomechanical processing).

- Analysis of pellet density and definition of motor step

Pellet-based extruders for AM require a preliminary calibration of motor steps to ensure the accurate fabrication of printed parts. Unlike filament-based AM, where the G-code ‘E’ value refers to the linear length of filament consumed, in pellet extrusion, ‘E’ corresponds to the screw rotation. This rotation must be precisely calibrated to deliver the mass of material required for a specific volume.

**Figure 3:**
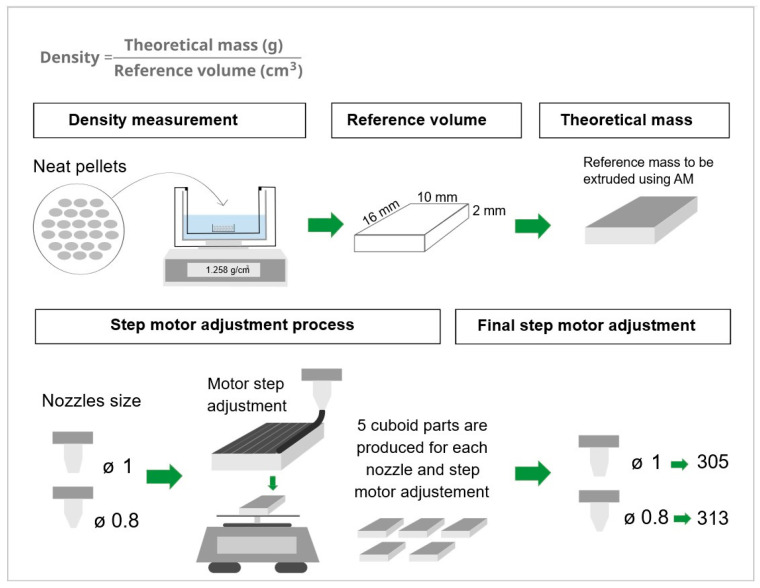
Characterization of pellet density and definition of motor step. First, the theoretical mass of the reference volume is obtained by measuring the density of neat PLA pellets. Second, the motor step for each nozzle is obtained by adjusting the mass of the cubic parts based on the theoretical mass.

To achieve this, a cuboid-shaped reference volume was defined (16 mm length × 10 mm width × 2 mm height), and the density of two materials in a pellet format, neat PLA and PLA-15% WOTP, was measured using an MDS 300 densimeter (Alfa Mirage Co., Ltd., Osaka, Japan) with alcohol as the displacement liquid. With this, the theoretical mass of the volume was calculated. Subsequently, five cuboid-shaped specimens were printed for each nozzle size, and the weight of each part was measured for neat PLA. This procedure was repeated iteratively (changing the motor steps) until the measured mass closely matched the theoretical mass, resulting in a value of motor steps of 305 and 313 for 1 and 0.8 mm nozzles, respectively (Figure 3).

- Analysis of flexural properties

To evaluate the effect of fiber content, nozzle size, and motor step setting on composite performance, the mechanical properties of flexural specimens were analyzed. At least 5 specimens per configuration were produced following the ISO 178 standard. Flexural testing was performed using a three-point bending setup, with a support span of 64 mm and a test speed of 2 mm/min. Tests were conducted on an LY-1065 machine (Dongguan Liyi Test Equipment, Dongguan, China).

- Density measurements in flexural test parts

The density of flexural test specimens was also analyzed in two ways: bulk density and true density. Bulk density was calculated from the weight of the specimens and their dimensions prior to mechanical testing, accounting for voids or porosity in the volume. True density was measured using the MDS 300 densimeter (Alfa Mirage Co., Ltd., Osaka, Japan). These measurements were performed to investigate differences between specimens printed with 1 mm and 0.8 mm nozzles and under two different motor step settings.

**Figure 4:**
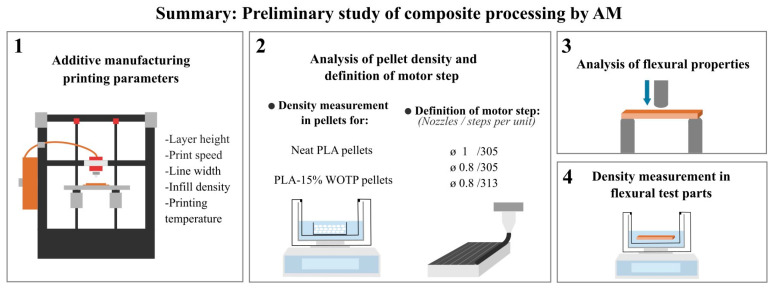
Summary: preliminary study of composite processing by AM. (1) Additive manufacturing printing parameters; (2) analysis of pellet density and definition of motor step; (3) analysis of flexural properties; (4) density measurement in flexural test parts.

Figure 4 outlines a summary of the preliminary study of composite processing in AM and the characterization of composites produced under different processing conditions.

### 2.6. Characterization Methods

#### 2.6.1. Fiber Dimensional Characterization

Fibers were characterized using a Morfi Compact morphological analyzer (Techpap SAS, Gieres, France), which analyzed the mean length-weighted fiber length and mean fiber width; these dimensions were measured following the standard ISO/FDIS 160652 [16] by the MorFi analyzer. Analyses were performed at three stages to monitor the evolution of fiber morphology during the composite processing: WTP and WOTP fiber before final milling and composite manufacturing, to characterize the initial fiber properties; WTP and WOTP fiber after pellet compounding, to evaluate the effect of extrusion and mechanical processing on fiber integrity; and WTP and WOTP fiber after final AM of the specimen, to assess potential fiber degradation or shortening induced during the AM process. All fiber measurements were carried out under the same conditions, except for the fiber WOTP before composite manufacturing, which was measured with an Olympus BX51 optical microscope (Olympus Corporation, Tokyo, Japan) due to the fiber obtained using the Morfi compact analyzer tending to give values lower than the actual size of the reinforcement. To extract the reinforcement from the composites, a Soxhlet apparatus with dichloromethane as the solvent was employed.

#### 2.6.2. Thermal Characterization

Thermogravimetric characterization (TGA) was conducted using a thermogravimetric analyzer (Mettler Toledo, Columbus, OH, USA) to evaluate the influence of the fiber on the polymer matrix and to assess the effect of the thermomechanical process. The temperature range was from 30 to 600 °C under a nitrogen atmosphere, with a heating rate of 10 °C/min. In addition, a differential scanning calorimetry (DSC) analysis was performed using a DSC822e calorimeter (Mettler Toledo, Columbus, OH, USA). Composite pellets were subjected to two heating cycles from 30 to 210 °C, with the first cycle used to eliminate the thermal history of the material.

#### 2.6.3. Rheology Measurements

Rheology measurements were performed using a modular compact rheometer MCR 302e (Anton Paar GmbH, Graz, Austria) equipped with a parallel-plate geometry and a 1 mm gap, operating in oscillatory mode. The frequency range was 0.1–100 Hz at a constant shear strain of 5%. Measurements were conducted at a constant temperature of 210 °C. The storage modulus (G′), loss modulus (G″), and complex viscosity (η*) were obtained.

#### 2.6.4. Scanning Electron Microscopy Characterization

After the flexural test, fracture surfaces were analyzed to assess the internal structure of the specimens and the fiber–matrix adhesion. Scanning electron microscopy (SEM) was performed using a model Tescan CLARA (TESCAN, Brno, Czech Republic), operating at an accelerating voltage of 1 kV and magnifications between 269× and 30,000×. Prior to observation, samples were sputter-coated with carbon.

#### 2.6.5. Mechanical Characterization

Flexural, tensile, and impact tests were performed in accordance with ISO 178, ISO 527-2, and ISO 180 [17,18,19], respectively. Flexural testing was carried out using a three-point bending setup with a support span of 64 mm and a test speed of 2 mm/min on an LY-1065 universal testing machine (Dongguan Liyi Test Equipment Co., Ltd., Dongguan, China). The same equipment was used for tensile testing, employing type 1AB specimens and a crosshead speed of 2 mm/min. For both tensile and flexural tests, the elastic modulus and maximum strength were determined. Izod impact tests were performed using an LY-XJJD 50 pendulum impact tester (Dongguan Liyi Test Equipment Co., Ltd., Dongguan, China). Specimens with dimensions of 80 × 10 × 4 mm were tested without a notch, with the impact direction perpendicular to the widest face. A minimum of five specimens were tested for each composite formulation and test, except for flexural and impact tests of WTP composites, for which four specimens were evaluated due to material constraints.

### 2.7. Statistical Analysis

Two separate statistical analyses were performed using MATLAB software (R2024b, MathWorks, Natick, MA, USA): the first evaluated the results of the preliminary studies (flexural properties and densities), and the second compared the mechanical properties of the compounds with and without thermomechanical processing. A confidence level of 95% was kept during the study. The data were evaluated by employing the Lilliefors test to analyze the normality. When the data fulfilled the normality assumptions (*p* > 0.05), the one-way analysis of variance (ANOVA) was employed to determine significant differences between groups, while when the normality was not accomplished, the Kruskal–Wallis test was performed. On the other hand, post hoc multiple comparisons were carried out employing the Tukey–Kramer test for ANOVA when significant differences were observed (*p* < 0.05), and for Kruskal–Wallis analysis, Dunn’s test with Sidak correction was utilized to identify pairs of differences among specific groups.

### 2.8. Summary of Manufacturing Processes and Characterization

**Figure 5:**
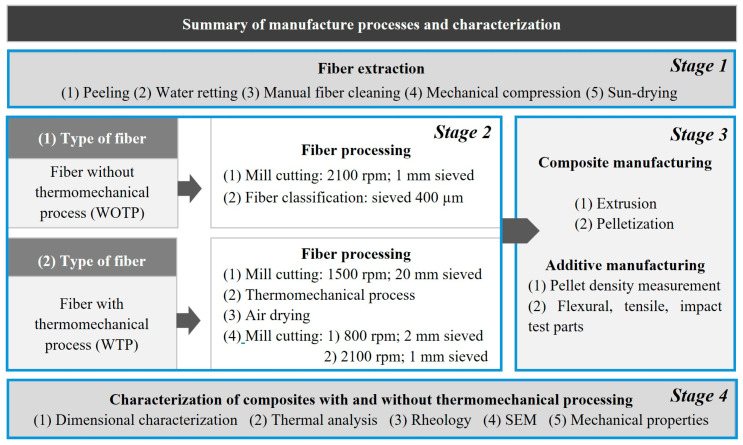
Summary of manufacturing processes and characterization: Stage 1: fiber extraction: the reinforcement is obtained through five stages; Stage 2: fiber processing: two types of fiber are produced; Stage 3: composites are manufactured; Stage 4: five characterization methods are used to analyze the materials.

A summary of the manufacturing processes is presented in Figure 5, showing the different steps involved in obtaining the fibers and composite materials.

## 3. Results and Discussion

### 3.1. Result of the Preliminary Study on Composite Processing by AM

- Results of pellet density and definition of motor step

The density of neat and PLA-15 wt.% WOTP pellets was determined as 1.258 ± 0.003 g/cm^3^ and 1.244 ± 0.002 g/cm^3^, respectively, the slight reduction being attributed to porosity generated during pellet manufacturing. Based on this value, the theoretical mass required (16 × 10 × 2 mm parts) for motor step calibration was calculated as 0.4026 g. Experimental results (Table 4) showed that the 1 mm nozzle with a motor step of 305 produced an average mass (0.4019 ± 0.0088 g) close to the theoretical value, indicating accurate extrusion. In contrast, the 0.8 mm nozzle with the same motor step resulted in under-extrusion (0.3897 ± 0.0080 g), while adjusting the motor step to 313 improved accuracy (0.4038 ± 0.0047 g) and repeatability. Although proper calibration was achieved for both nozzle sizes, the 1 mm nozzle was selected for subsequent experiments due to its larger diameter, which facilitates material flow and reduces the risk of clogging when processing fiber-reinforced composites (Table 4).

- Results of flexural properties

**Figure 6:**
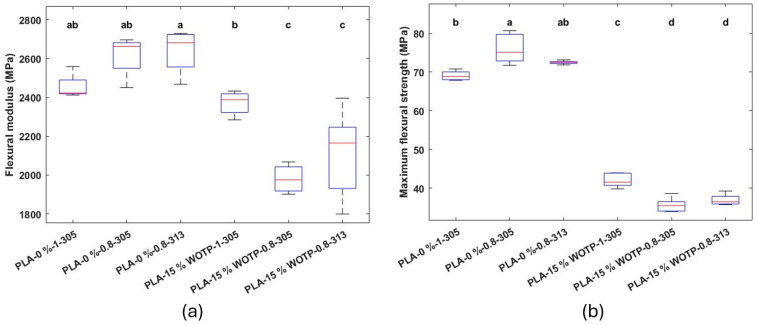
Preliminary study of flexural properties of the different groups, identified by material composition, nozzle diameter, and motor steps (letters indicate statistically similar groups, ‘a’ being the groups with the highest values and ‘d’ the groups with the lowest values): (**a**) flexural modulus; (**b**) maximum flexural strength.

A general reduction in flexural performance was observed in reinforced samples compared to unreinforced ones under identical processing conditions (except for the 1 mm nozzle in elastic modulus, where no statistical differences were found between reinforced and unreinforced materials). This behavior can be attributed to insufficient interfacial adhesion between the reinforcement and the polymer matrix (Figure 6).

On the other hand, no significant differences were observed between the same material processed with the same nozzle diameter and different motor steps, thus concluding that, from a mechanical point of view, the adjustment of the motor steps was not relevant. However, this adjustment was essential to ensure accurate material deposition and reduce variability. In contrast, the nozzle diameter influences mechanical results. While the 0.8 and 1 mm nozzles performed similarly for unreinforced PLA, the 1 mm nozzle provided superior flexural strength and elastic modulus for the fiber-reinforced compounds. Additionally, the 1 mm nozzle offers a more robust processing window for fiber-reinforced materials, minimizing clogging risk and ensuring stable extrusion, thus being, as commented before, the preferred option for subsequent experiments.

- Results of density measurements in flexural test parts

**Figure 7:**
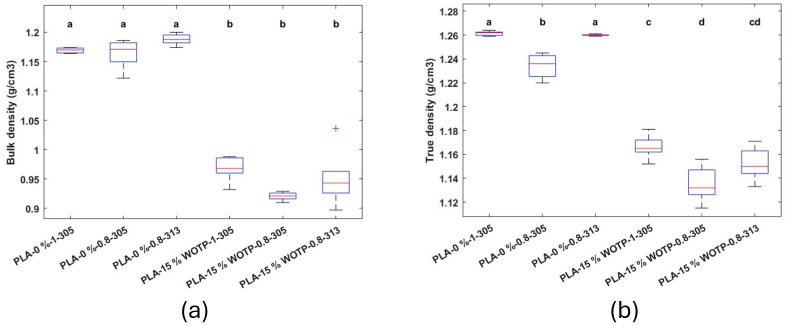
Density measurements of flexural test parts (letters indicate statistically similar groups, with ‘a’ being the groups with the highest values and ‘d’ the groups with the lowest values): (**a**) boxplot of bulk density; (**b**) boxplot of true density.

Figure 7 presents the density measurements of flexural specimens, including both true density and bulk density. A reduction in bulk density compared to true density is observed, which can be attributed to the presence of internal voids within the material. In AM samples, the addition of natural fibers leads to an increase in porosity. This behavior has been previously reported by Xiao et al., who attributed it to the increase in melt viscosity [20], which reduces flow through the nozzle and results in poor interlayer adhesion. Furthermore, the results show a decrease in density values when rachis fiber is incorporated into the matrix. When samples produced under the same processing conditions are compared, for example, composites with 0 wt.% and 15 wt.% fiber using a 1 mm nozzle and a motor step of 305, the bulk density decreases from 1.169 to 0.967 g/cm^3^, while the true density decreases from 1.261 to 1.166 g/cm^3^. In addition, bulk density shows no significant differences between parts with the same groups of material, whereas for true density, the specimen produced with 0 wt.% fiber and with a 0.8 mm nozzle (305 steps) shows significant differences (lower values) compared to the other two parts without fiber. The same occurs when 15 wt.% fiber is added, obtaining significant differences between parts with 1 and 0.8 mm nozzles with a step motor of 305. The reduction in density when the step motor is not adjusted highlights the importance of this process during initial AM stages.

### 3.2. Characterization of Composites with and Without Thermomechanical Processing

#### 3.2.1. Results of the Dimensional Characterization of the Fiber

**Figure 8:**
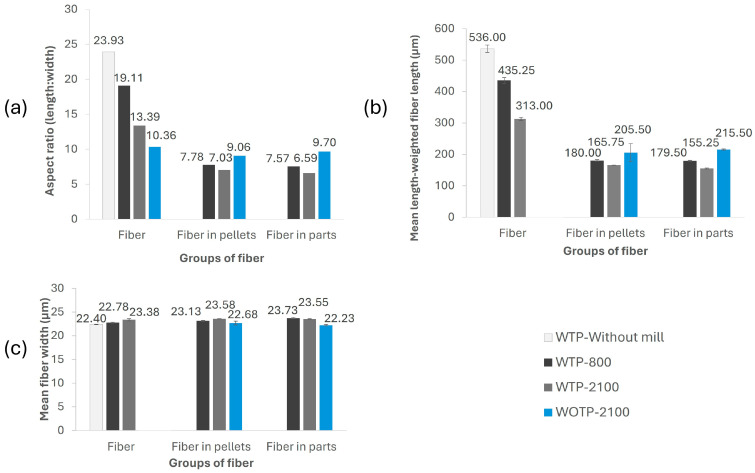
Fiber dimensional measurements: (**a**) aspect ratio (length/width); (**b**) mean length-weighted fiber length; (**c**) mean fiber width.

In Figure 8a, the results of the dimensional characterization of the fiber are shown, including the fiber length and width for each WTP and WOTP composite. The highest aspect ratio (length/width) is observed in fibers subjected to TP prior to final milling, reaching a value of 23.93. When the milling process is performed at 800 rpm, the aspect ratio decreases to 19.11 and further decreases to 13.39 at 2100 rpm. In contrast, fibers without TP exhibit an even lower aspect ratio, reaching 10.36 at 2100 rpm.

On the other hand, a decrease in the aspect ratio is observed when the fiber is processed in pellet form due to the inherent cutting process, resulting in ratios of 9.06 for WOTP fibers, 7.03 for WTP-2100 fibers, and 7.78 for WTP-800 fibers. The aspect ratio observed in AM parts is similar to that found in pellets, thus concluding that the MEX AM process does not affect this factor. Regarding dimensional measurements, the fiber width remains similar across the three processing stages, ranging from 22.23 to 23.73 µm (Figure 8c). However, noticeable differences are observed in fiber length before and after the pelletized process, with values ranging from 536 to 313 µm before composite manufacturing and decreasing further from 180 to 165.75 µm afterward for WTP fiber. In contrast, WOTP fiber before the cutting process had a length of 2554.83 µm and a width of 246.65 µm; these results are not included in Figure 8b,c in order to maintain a clear and consistent scale on the axes, while after the pelletized process, fiber size was reduced to a length of 206 µm and a width of 22.7 µm. Therefore, WTP fiber suffered a further length decrease during the compound manufacturing, resulting in a lower aspect ratio compared to WOTP fiber. This lower aspect ratio was also observed in final printed parts.

The reduction in fiber length is directly related to the processability of the materials during the manufacturing process of the composite. Furthermore, an increase in fiber content impacts both fiber size and the melt viscosity of the composite. During the compounding process, shear forces occur, which are consequently determined by the melt viscosity [21]. According to Peltola et al., to control the viscosity of the material and, therefore, the fiber size, a plasticizer could be utilized to control the viscosity. The use of the plasticizer reduces the energy absorbed by the compound when the blending is performed [21], thus repercussing in the fiber length.

In addition, You et al. analyzed the aspect ratio of wood fiber treated with a hydrothermal process and obtained a reduction in the reinforcement size after the treatment, resulting from the deterioration of unsteady hemicellulose and the disruption of fiber cell walls during the hydrothermal process [22]. Therefore, the reduction in the aspect ratio of WTP fibers in pellets compared to WOTP fibers could be due to a change in the inner structure of the reinforcement, caused by a decrease in the fiber components following the hydrothermal process, such as hemicellulose [22], which serves as a bridge between the lignin and cellulose [23]. During the hydrothermal treatment, a hydrolysis process and the detachment of hemicellulose from within the cell walls occur. This, along with the redistribution of the lignin during the process, causes the collapse and deformation of the cell walls [22], which likely results in the reduction in aspect ratio of the reinforcement.

#### 3.2.2. Results of the Thermogravimetric Analysis

Three groups of compounds were analyzed in pelletized form: a composite with WOTP-2100 fiber, a composite with WTP-2100 fiber and a composite with WTP-800 fiber. The temperatures analyzed were obtained with the intersection of extrapolated starting mass and end mass, with the tangent applied to the maximum slope of the TGA curve.

Processing temperature is a critical factor in composite fabrication. During processing, different thermal ranges are employed, such as the pellet extrusion or the additive manufacturing process, both of which could affect the final properties of the material. The temperatures utilized for both neat PLA and composites remain within the thermal stability limits of the compounds. However, other research, such as the study by Kostenko et al., analyzes that the addition of a different component to the matrix may affect the degradation of the material. They found that the use of (3-aminopropyl)triethoxysilane (APTES) can induce thermal degradation through various chemical and physical mechanisms. In addition, that study conducted a spectroscopy analysis (FTIR) and concluded that the use of APTES pointed to the formation of low-molecular-weight degradation products [24].

**Figure 9:**
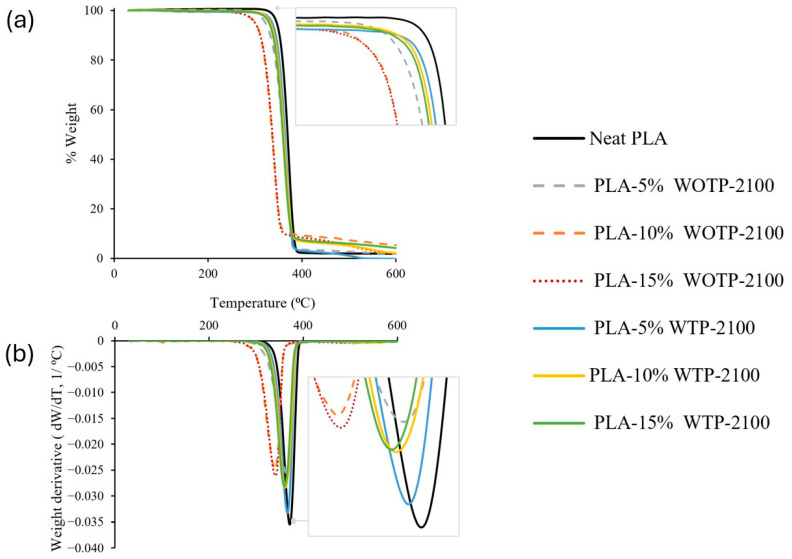
Thermogravimetric analysis of pellet composites (5, 10, 15 wt.%), with and without thermomechanical processing, using a fiber milling setting of 2100 rpm: (**a**) TGA analysis; (**b**) DTG analysis.

**Figure 10:**
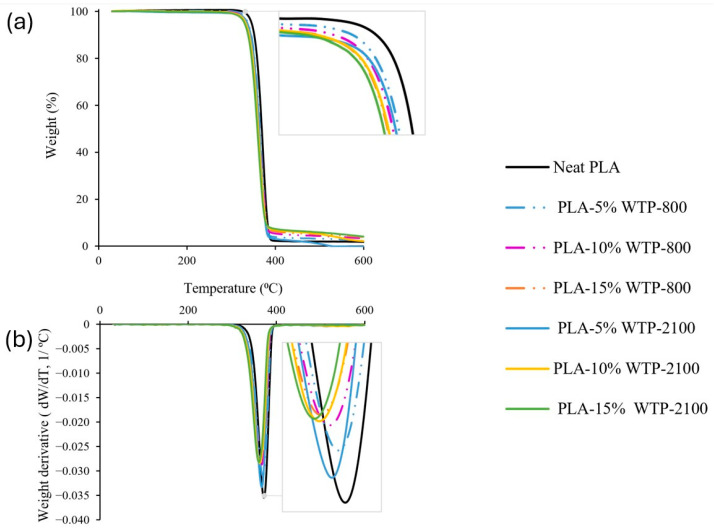
Thermogravimetric analysis of pellet composites (5, 10, 15 wt.%) with thermomechanical processing, using a fiber milling setting of 800 rpm and 2100 rpm: (**a**) TGA analysis; (**b**) DTG analysis.

In general, a reduction in thermal stability is observed upon the addition of natural fibers, as is shown in Table 5 and Figure 9 and Figure 10. This is attributed to the lower degradation of the natural fibers compared to the polymer matrix. It is observed that the first group (WOTP-2100) exhibits the lowest onset temperature (Tonset) range, with temperatures between 337.53 °C and 314.80 °C. In contrast, the range for the second group (WTP-2100) is 347.52 °C and 340.52 °C, corresponding to 5% and 15% fiber, respectively. This difference among composites with and without TP occurs because the application of a water-based process involving specific temperature and conditions leads to the reduction of components such as waxes and impurities [8]. Consequently, the Tonset variations observed between the different composites are attributed to this removal. Similar temperatures were noted for the second and third groups, with a difference of approximately 2 to 3 °C higher for the group with the larger reinforcement dimension (WTP-800).

On the other hand, endpoint temperatures (Tendpoint) show similar values in the second and third groups with a difference of approximately 2 to 3 °C within groups, the ranges being between 381.83 °C and 374.79 °C. The lowest registered Tendpoint is for composites with 15% WOTP fiber with 352.77 °C. In addition, similar results occur for composite materials regarding the peak temperature of the derivative TGA (Tpeak DTG), with ranges between 368.66 °C and 363 °C. The data shows the same value for the second group with 10% and 15% fiber and for the third group with 15% fiber with a temperature of 363 °C.

#### 3.2.3. Results of the Differential Scanning Calorimetry Analysis

**Figure 11:**
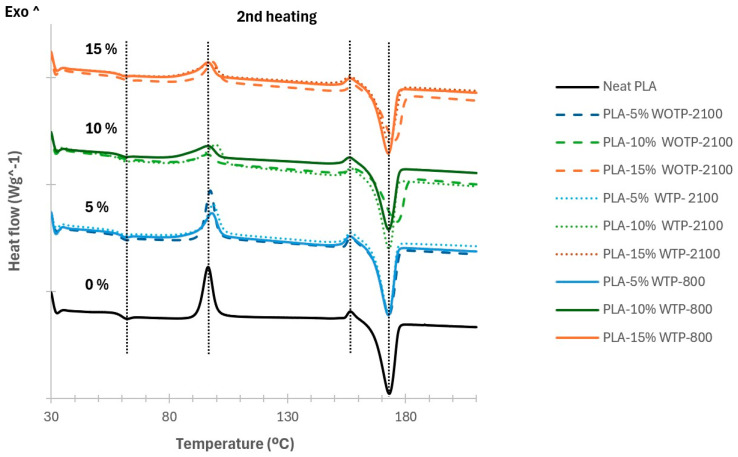
DSC analysis of the second heating cycle for the composite in pellet format: with and without thermomechanical processing. The dotted vertical lines indicate the temperature changes of the composites compared to neat PLA.

Table 6 and Figure 11 present the DSC thermal analysis of the second heating cycle for the composite in pellet format, with and without thermomechanical processing. The glass transition temperature (Tg) and the peak melting temperature (Tpm) are similar to the specifications of the manufacturer for neat PLA (60 °C and 175 °C, respectively); the analysis obtained was a Tg onset of 57.71 °C and a Tpm of 172.48 °C. Moreover, the material showed an exothermic crystallization (Tc-1) at 96.43 °C. The results obtained for the matrix are similar to findings in the literature [25,26]. A small exothermic peak (Tc-2) before the melting peak point is observed at 156.57 °C; according to the study described in Ke and Sun [27], this peak is due to an additional crystallization.

On the other hand, different formulations of composites are analyzed; Tg and Tpm have similar results for all groups of composites, with a Tc-2 peak of 156 °C and a Tpm peak of 172 °C. Except for composite fiber without TP, which shows an increase of 3 °C in Tpm peak for 10% and 15% fiber, with peaks of 175.74 °C and 174.94 °C, respectively. In addition, for both materials with TP, it can be noted that when the fiber size grows (WTP-800), the melting point temperature decreases. In accordance with Basiji et al., this occurred because smaller fibers interact more forcefully with the matrix material [28].

For composites without TP and with TP with a shorter fiber length setting (WTP-2100), the glass transition onset temperature has a similar value of 57 °C. For the material with TP with a longer length fiber configuration (WTP-800), the Tg onset is 1 °C less.

#### 3.2.4. Results of the Rheology Characterization

**Figure 12:**
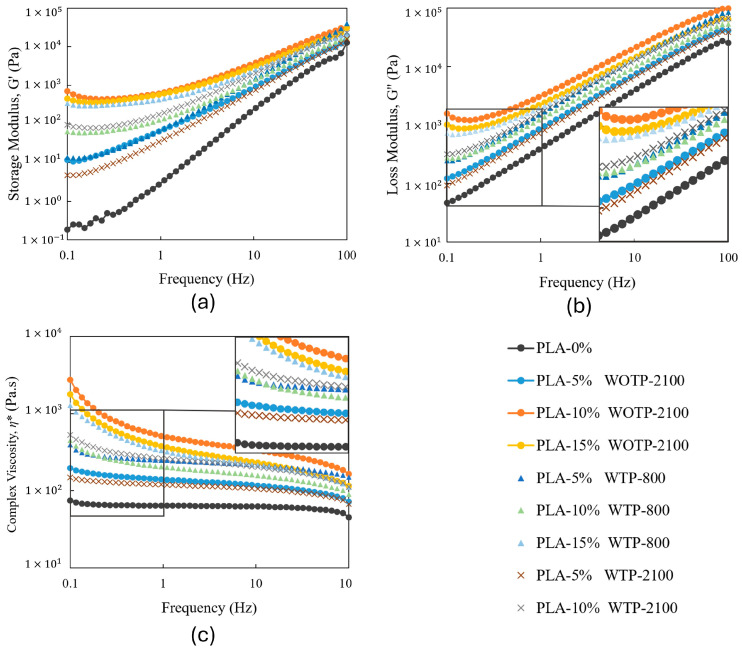
Rheology characterization: (**a**) storage modulus; (**b**) loss modulus; (**c**) complex viscosity.

Figure 12a–c shows the frequency dependence of the storage modulus (G′), loss modulus (G″), and complex viscosity (η*) for all the investigated composites. Both G′ and G″ exhibit a pronounced dependence on frequency across the entire range, which is characteristic of viscoelastic polymer systems.

For neat PLA, a predominantly liquid-like behavior is observed, particularly at low frequencies, where G″ exceeds G′ (Figure 12a). As the frequency increases, both moduli increase, although the response remains dominated by viscous contributions. In contrast, the incorporation of rachis fibers leads to a significant increase in G′ and a progressive reduction in the slope of its frequency dependence. This behavior indicates a transition towards a more elastic-dominated response, which becomes more pronounced as the fiber percentage increases.

The enhanced elastic behavior of the composites can be attributed to restricted polymer chain mobility caused by filler–matrix interactions, as well as to the development of a network-like structure within the material. Such behavior is typically associated with the formation of a percolated fiber network, which imparts solid-like characteristics to the melt even at low frequencies [29,30].

Differences between the composite groups are also observed as a function of processing conditions and fiber characteristics. In general, WOTP composites exhibit higher G’ values and a more pronounced elastic response compared to WTP systems over the entire frequency range. This suggests a more effective structural organization in WOTP materials. In contrast, WTP composites show a comparatively lower elastic contribution, which may be related to differences in fiber size, morphology, and dispersion within the matrix.

The complex viscosity results (Figure 12c) further support these observations. All composites show higher η* values than neat PLA, confirming the increased resistance to flow induced by fiber addition. Moreover, WOTP systems consistently exhibit higher viscosity than WTP composites. This behavior may be related to differences in fiber size and morphology, where smaller or less interactive structures in WTP materials may reduce effective fiber–fiber interactions and network connectivity within the melt, leading to lower viscosity values [31]. This behavior suggests a higher degree of structural connectivity and resistance to flow in WOTP systems. For WTP, a lubrication-like effect may be considered, where the morphology and distribution of the reinforcement facilitate polymer chain mobility and reduce internal friction. However, this effect should be interpreted cautiously, as it is likely coupled with differences in dispersion state and structural organization rather than representing a purely lubricating mechanism.

**Figure 13:**
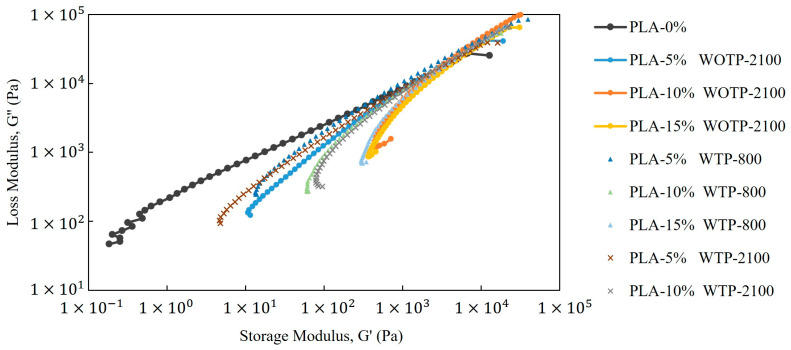
Cole–Cole representation (loss modulus, G″ vs. storage modulus, G′).

Cole–Cole plots (G″ vs. G′) were used to evaluate the homogeneity of the systems and to identify potential phase separation phenomena (Figure 13). The neat PLA sample exhibits a relatively smooth and continuous curve, indicating a more homogeneous viscoelastic response with a narrower relaxation time distribution. In contrast, all composite systems show clear deviations from the ideal semicircular behavior, including distortions, extended tails, and changes in curvature. These features indicate the presence of multiple relaxation mechanisms and structural heterogeneities, which can be attributed to filler–matrix interactions and the development of complex internal structures.

The observed deviations from ideal behavior suggest that the incorporation of fibers introduces a broad distribution of relaxation times, consistent with heterogeneous microstructures and possible localized phase separation or aggregation effects. Furthermore, WOTP composites show more pronounced distortions than WTP systems, indicating a higher degree of structural complexity.

**Figure 14:**
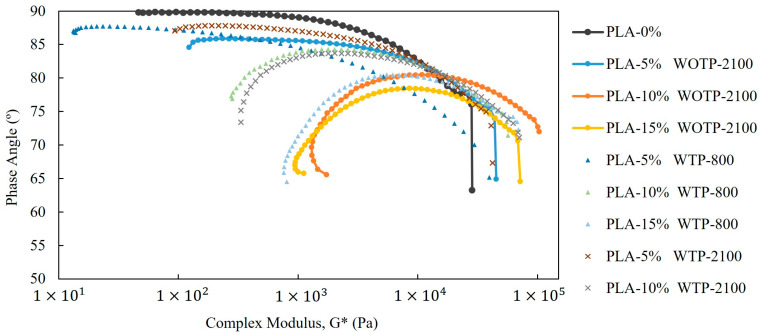
Van Gurp–Palmen representation of the phase angle (°) as a function of the complex modulus (G*) for neat PLA and the corresponding composites.

The Van Gurp–Palmen representation (phase angle as a function of complex modulus G*) provides further insight into the viscoelastic behavior and structural evolution of the materials (Figure 14). The neat PLA sample exhibits high phase angles at low complex modulus values, followed by a sharp decrease with increasing modulus, which is characteristic of a predominantly viscous response transitioning towards elastic behavior at higher frequencies.

In contrast, all composite systems display significantly lower phase angles and a markedly reduced dependence on complex modulus. This indicates a more elastic-dominated response across the entire range and reflects restricted polymer chain mobility due to the presence of fibers. The progressive flattening of the curves and the absence of high phase angle regions suggest the formation of a percolated fiber network within the composites. This behavior is particularly evident in WOTP materials, which show lower phase angles and a stronger deviation from the neat polymer response compared to WTP systems.

Overall, the comparison between WOTP and WTP composites reveals that processing conditions and fiber characteristics play a critical role in determining the rheological behavior of the materials. WOTP systems tend to develop a more interconnected and elastic network structure, leading to higher modulus and viscosity values, whereas WTP systems exhibit a less developed structure with a relatively higher viscous contribution. These differences highlight the competition between network formation and lubrication-like effects, which ultimately control the viscoelastic response of the composites.

#### 3.2.5. Results of the Scanning Electron Microscopy Characterization

**Figure 15:**
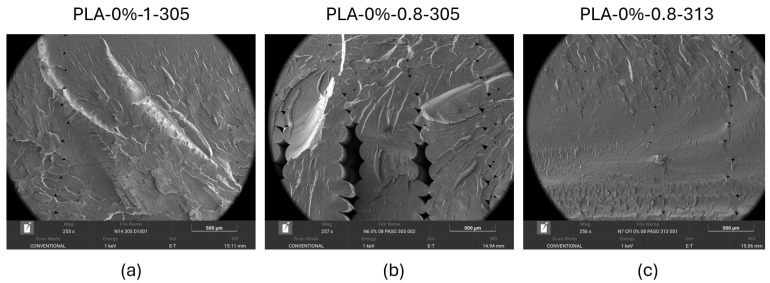
SEM characterization of flexural test samples with 0% fiber content: (**a**) diameter 1-step 305; (**b**) diameter 0.8-step 305; (**c**) diameter 0.8-step 313.

The fracture zone in the flexural test samples was analyzed using a scanning electron microscope (SEM). The following parameters were analyzed: fiber percentage (0%, 5%, 10% and 15%) for both compounds (WTP and WOTP), nozzle size (1 and 0.8 mm) and motor steps (305 and 313). In Figure 15a,c, it is observed that for the 0% fiber flexural parts, the motor step has been adjusted according to the type of material and the nozzle size. The resulting adjusted motor steps were 305 for the 1.0 mm nozzle and 313 for the 0.8 mm nozzle. This small change in the setting led to an increase in the amount of material extruded during the process, resulting in a greater homogeneity in the deposition of the material. Consequently, a visible reduction in voids is observed in the internal structure compared to the sample where the motor step has not been adjusted, as shown in Figure 15b. In addition, the gaps in the unadjusted flexural part explain the reduction in the results presented in the preliminary study of the density, where there were significant differences between the true density for the adjusted and unadjusted parts for the 0.8 mm nozzle. Furthermore, these voids could be the cause of an increase in the dispersion of data in tensile strength in the preliminary flexural test.

**Figure 16:**
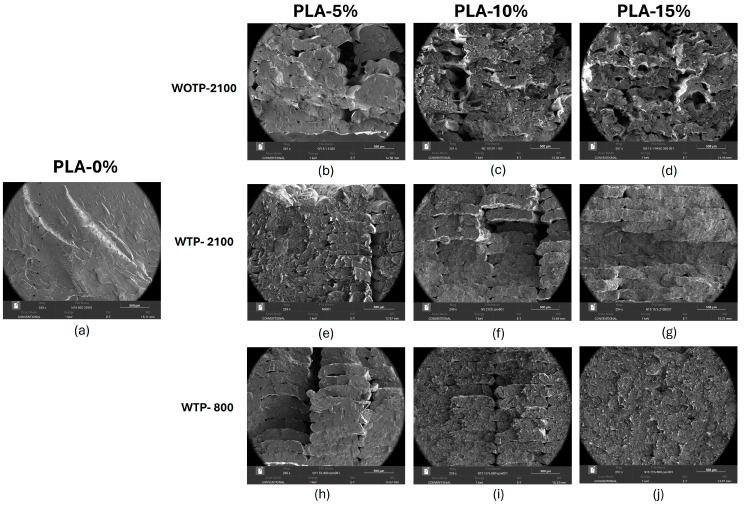
SEM characterization of flexural test samples with 0%, 5%, 10%, and 15% fiber content with and without TP: (**a**) neat PLA; (**b**–**d**) composites without TP, using a fiber milling setting of 2100 rpm; (**e**–**g**) composites with TP, using a fiber milling setting of 2100 rpm; (**h**–**j**) composites with TP, using a fiber milling setting of 800 rpm.

It is noted that the addition of fiber causes a growth in the inner porosity of test parts, which reduces the homogeneity of the structure of filament extruded [20]. Comparing flexural test parts with and without TP, WTP samples showed a reduction in voids between filaments and a better definition of extruded material than WOTP test parts (Figure 16). This is due to a better flow of material during the additive manufacturing process, caused by the reduction of viscosity.

**Figure 17:**
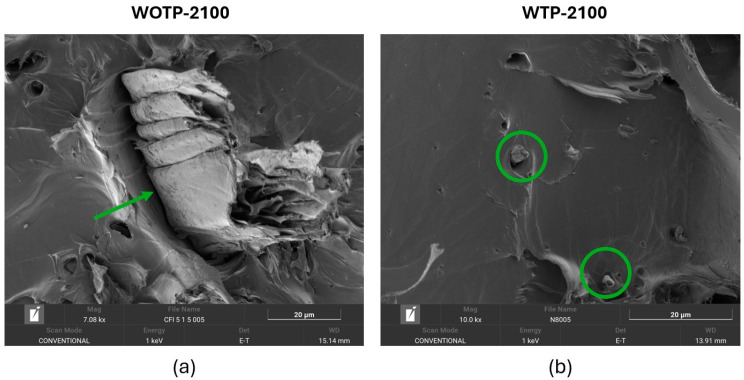
SEM characterization of flexural test samples: (**a**) WOTP-2100 composite: the green arrow indicates a higher content of fiber clusters in the compound; (**b**) WTP-2100 composite: green circles show individual fibers in the composite.

On the other hand, in Figure 17 the morphology of fiber is analyzed. It is observed that the WOTP composite possesses a higher content of clusters of fiber with larger dimensions, as shown in Figure 17a, while a reduction in sizes and the individualization of fiber are contemplated in Figure 17b for the WTP composite.

**Figure 18:**
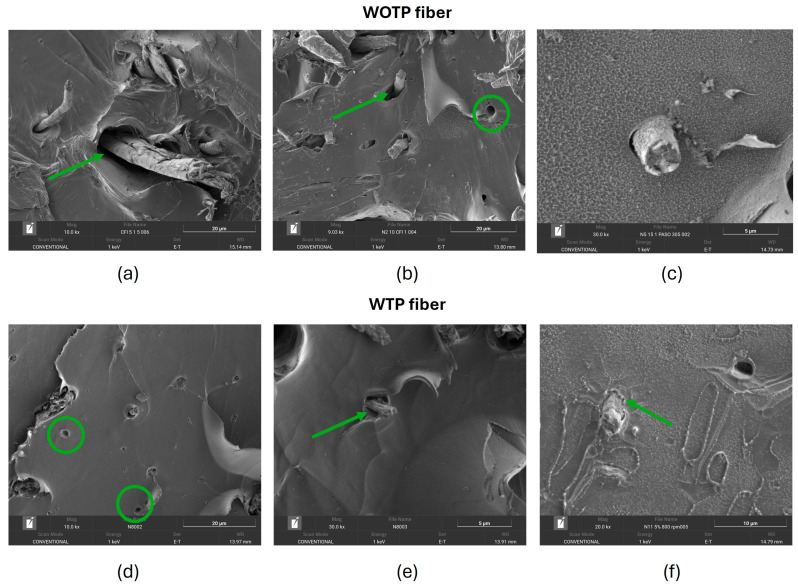
WOTP fiber: (**a**) voids matrix–fiber; (**b**) pull-out; (**c**) voids matrix–fiber. WTP fiber: (**d**) pull-out; (**e**) voids matrix–fiber; (**f**) adherences matrix–fiber. The green circles indicate the voids left by the fiber, and the arrows indicate the voids between the fiber and the matrix or their adhesion.

In both cases, it is noted that sliding of the fiber through the matrix occurs (fiber pull-out); Figure 18b,d shows holes left by the fibers when they were detached from the polymer. In addition, Figure 18a–c,e show voids between matrix and reinforcement, which is common in natural-fiber composites [32,33]. This occurs because the hydrophilicity of natural fibers and the hydrophobicity of polymeric materials result in a reduction of interfacial bonding between the two elements. This leads to poor stress distribution and the development of voids. A method to enhance the interfacial adhesion considering the hydrophilicity of the fiber is hydrothermal treatment, which modifies the reinforcement structure [8]. One approach to improving this adhesion is to provide a better mechanical interlocking of the matrix to the fiber surface, achieved by modifying the surface of the reinforcement. Oliveira Camillo et al. explain that performing the hydrothermal process on the fiber removes the impurities or waxes from the reinforcement, showing a rougher surface that favors better physical anchoring [8]. Figure 18f shows a slight improvement in interfacial adhesion in composites with TP compared to compounds without TP, where the PLA matrix is incorporated into the irregularities of the fiber.

#### 3.2.6. Results of the Mechanical Characterization

For mechanical testing, the TP compound milled at 800 rpm was used, as it preserved a higher fiber aspect ratio than the 2100 rpm samples.

In general, composites manufactured with TP obtained higher results than composites without TP. This is due to the result of using a material with lower viscosity and better flow (caused by a reduction in fiber size), which allows a better flow. In addition, the hydrothermal treatment enhances the adherence between the matrix and fiber; the fiber surface becomes rough as a result of the treatment [22], producing fewer voids between layers during the additive manufacturing process, resulting in higher mechanical properties.

**Figure 19:**
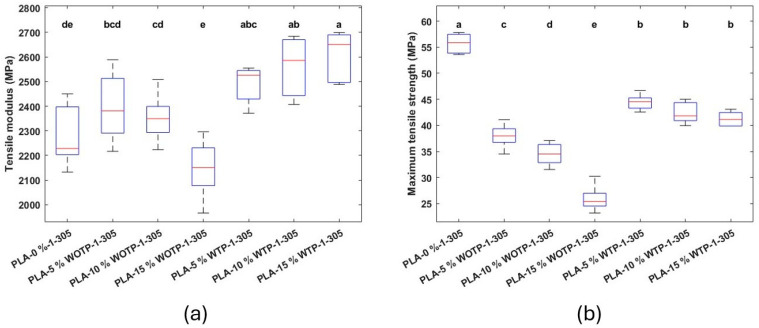
Composite with 0, 5, 10, and 15% fiber: (**a**) tensile modulus; (**b**) maximum tensile strength. Letters indicate statistically similar groups, with ‘a’ being the groups with the highest values and ‘e’ the groups with the lowest values.

Firstly, tensile modulus statistically increases with respect to the matrix when the material with TP is employed, from 2273.54 ± 123.66 MPa (neat PLA) to 2612.51 ± 95.16 MPa (PLA-15% WTP). In addition, it is observed that for 5 and 10% of WOTP fiber, mean values increase compared to neat PLA (still with no significant differences) but clearly decrease with 15% fiber content. The addition of reinforcement to the neat PLA reduces the tensile strength for both groups of compounds. The increase in fiber content produces a significant reduction in the tensile strength when fiber without TP is used, from 55.74 ± 1.84 MPa (neat PLA) to 25.92 ± 2.25 MPa (PLA-15% WOTP). The tensile strength for composites with treatment shows no significant differences regardless of the fiber content but presents statistically higher values than compounds without treatment. Overall, results show a reduction in tensile strength for both composites with and without TP with respect to the matrix. However, WTP compounds achieved a higher tensile strength than WOTP compounds. This is consistent with different studies, in which the hydrothermal process applied to the fiber reduces the amount of extractive material found in the fiber, leading to a roughened fiber surface morphology. This improves the adherence to the matrix and, therefore, the properties [8,23] (Figure 19).

**Figure 20:**
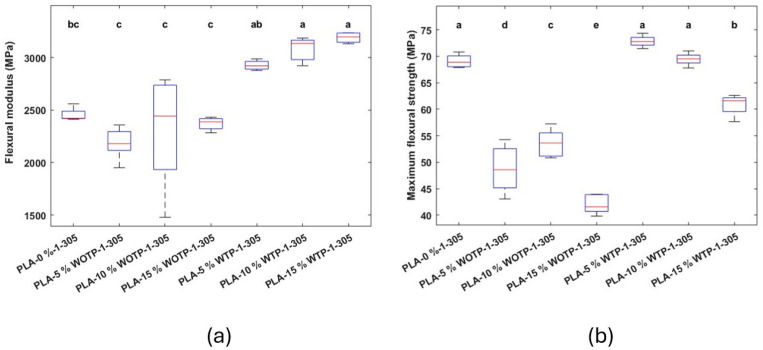
Composite with 0, 5, 10, and 15% fiber: (**a**) flexural modulus; (**b**) maximum flexural strength. Letters indicate statistically similar groups, with ‘a’ being the groups with the highest values and ‘e’ the groups with the lowest values.

On the other hand, the addition of reinforcement with TP to the matrix significantly increased the flexural modulus from 2456 ± 61.16 MPa (neat PLA) up to 3189.68 ± 52.24 MPa (PLA-15% WTP). In the case of compounds without TP, no significant differences were observed compared to neat PLA. Regarding flexural strength, TP compounds slightly increased the values compared to neat PLA when 5 and 10% of TP reinforcement were used (still with no significant differences) but statistically decreased for 15% TP reinforcement. In contrast, when fiber without TP was employed, the flexural strength was significantly reduced below the PLA and the composites with thermomechanical processing. The decrease in flexural properties with the addition of natural fibers, when no treatment is used, aligns with the existing literature [34,35]. The reduction in properties occurred due to an insufficient adhesion between matrix and filler, as observed in SEM characterization when the pull-out effect takes place (Figure 20).

**Figure 21:**
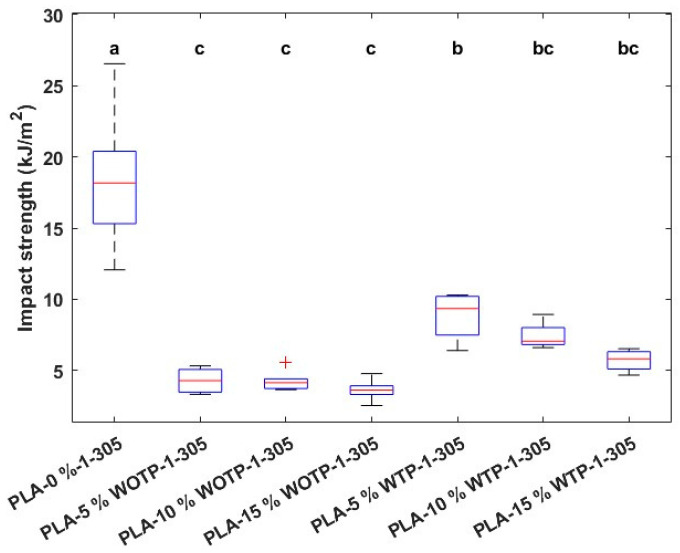
Impact strength in composites with 0, 5, 10, and 15% fiber. Letters indicate statistically similar groups, with ‘a’ being the groups with the highest values and ‘c’ the groups with the lowest values.

Finally, impact strength shows a decrease in properties for both WTP and WOTP composites compared to neat PLA. There are no significant differences in values among the compounds without TP, while composites with TP achieved higher results compared to composites without TP, but only with statistical significance for 5% WTP fiber (Figure 21). This improvement occurs due to better adhesion between the matrix and reinforcement for the composite with TP, caused by a rougher surface that favors better physical anchoring. Whereas results show an improvement for 5% WTP fiber, compared to the composite without TP, a noticeable reduction occurs when rachis fiber is added to the matrix. This could be due to inadequate adhesion between the elements, causing the reinforcement to behave as a site of stress concentration, generating micro-voids between the matrix and the fiber. When the impact test is produced, these micro-voids promote the crack propagation and, therefore, reduce the impact strength of the material [35].

## 4. Conclusions

In this study, the characterization of a biodegradable composite based on PLA and rachis fiber in pellet format was evaluated using an additive manufacturing process. The main conclusions are as follows:

Preliminary results show that the density of neat PLA and PLA-15 wt.% WOTP pellets is 1.258 ± 0.003 g/cm^3^ and 1.244 ± 0.002 g/cm^3^, respectively. This reduction in density is associated with the porosity generated during the pellet manufacturing. Concerning the flexural test, a noteworthy reduction in flexural properties is observed when 15 wt.% fiber is employed in parts with two nozzles and two motor step configurations. The step motor configuration used during part production affects the final mass and density of the samples.

The change in fiber dimensions shows how the reinforcement size depends on the manufacturing processes. The WTP fiber exhibits an aspect ratio of 23.93 before milling, which decreases to 7.78 after compounding (WTP-800). However, WOTP fiber suffers a lower reduction in aspect ratio in the compounding process (from 10.36 before compounding to 9.06 measured in pellets), obtaining a higher aspect ratio both in the pellets and the final printed parts.

The storage and loss moduli present a dependence on frequency for all studied materials, with composites exhibiting increasingly elastic-like behavior as fiber content rises. WOTP compounds show a more pronounced elastic melt response, reflected in higher storage modulus values compared to WTP materials, regardless of fiber size. In general, the incorporation of reinforcement leads to an increase in complex viscosity. This behavior is further supported by the reduction in phase angle, indicating restricted chain mobility and the progressive development of a more structured viscoelastic response.

The internal structure of the flexural samples, as revealed by SEM analysis, is significantly influenced by the initial motor step adjustment. When comparing adjusted motor steps with unadjusted ones, the latter exhibits voids between the extruded filaments. The addition of fiber increases the internal porosity of the specimens. Furthermore, when comparing WOTP and WTP flexural samples, WTP specimens show reduced voids between matrix and reinforcement compared to WOTP specimens.

The mechanical properties of the composites present higher average values for WTP compounds compared to WOTP materials due to reduced viscosity and the improved matrix–fiber and interlayer adhesion during the additive manufacturing process (and despite the lower aspect ratio of the WTP fiber compared to the WOTP in the printed parts). The lower properties observed in WOTP composites are attributed to fiber pull-out at the fiber–matrix interface and increased internal porosity in the manufactured parts. Furthermore, the addition of WTP fiber to PLA significantly increases the tensile modulus, from 2273.54 ± 123.66 MPa (neat PLA) to 2612.51 ± 95.16 MPa (PLA-15% WTP). A similar trend was observed for the flexural modulus, from 2456 ± 61.16 MPa for neat PLA to 3189.68 ± 52.24 MPa for PLA-15% WTP.

## Figures and Tables

**Table 1:** Composites with three fiber contents (5, 10, and 15 wt.%); composite groups: (1) WOTP fibers with a milling setting of 2100 rpm; (2) WTP fibers with a milling setting of 2100 rpm; (3) WTP fibers with a milling setting of 800 rpm.

Composite Groups	
(1)	PLA-5% WOTP-2100
	PLA-10% WOTP-2100
	PLA-15% WOTP-2100
(2)	PLA-5% WTP-2100
	PLA-10% WTP-2100
	PLA-15% WTP-2100
(3)	PLA-5% WTP-800
	PLA-10% WTP-800
	PLA-15% WTP-800

**Table 2:** Components and physical properties of the PLAL105 pellets.

Biobased content	100%
Stereochemical purity	≥99% (L-isomer)
Residual monomer	≤0.45%
Melting temperature	175 °C
Glass transition temperature	60 °C

**Table 3:** Additive manufacturing printing parameters.

Layer height (mm)	0.2	Printing temperature (°C)	217
Initial layer height (mm)	0.2	Build plate temperature (°C)	60
Line width (mm)	1	Initial layer speed (mm/s)	10
Wall line count	3	Print speed (mm/s)	15
Infill density (%)	100	Travel speed (mm/s)	50
Infill pattern	Parallel lines		

**Table 4:** Cuboid-shaped mass measurement for two different nozzle diameters (1 and 0.8 mm) and motor steps in neat PLA (theoretical mass value of 0.4026 g).

	Nozzle Diameter (mm)/Steps per Unit		
Specimens	1/305	0.8/305	0.8/313
Mass 1 (g)	0.4015	0.3809	0.4042
Mass 2 (g)	0.4030	0.3832	0.3989
Mass 3 (g)	0.4129	0.3942	0.4115
Mass 4 (g)	0.4035	0.4004	0.4017
Mass 5 (g)	0.3884	0.3897	0.4027
**Mean (g)**	0.4019	0.3897	0.4038
**Standard deviation (g)**	0.0088	0.0080	0.0047
**Mean percentage error vs. theoretical value (%)**	−0.17	−3.20	0.30

**Table 5:** TGA analysis for three groups of materials in pellet form: (1) composite: WOTP with a fiber milling of 2100 rpm; (2) composite: WTP with a fiber milling of 2100 rpm; and (3) composite: WTP with a fiber milling of 800 rpm.

	Groups of Composites	T_onset_ (°C)	T_endpoint_ (°C)	T_peak DTG_ (°C)
	Neat PLA	354.08	383.17	372.50
(1)	PLA-5% WOTP-2100	337.53	378.43	366.16
	PLA-10% WOTP-2100	314.80	353.17	339.89
	PLA-15% WOTP-2100	316.41	352.77	342.00
(2)	PLA-5% WTP-2100	347.52	378.20	366.67
	PLA-10% WTP-2100	342.75	376.79	363.00
	PLA-15% WTP-2100	340.52	374.79	363.00
(3)	PLA-5% WTP-800	349.26	381.83	368.66
	PLA-10% WTP-800	345.47	379.58	366.33
	PLA-15% WTP-800	342.57	377.43	363.00
	**Minimum Tonset**	314.80	352.77	339.89
	**Maximum Tonset**	354.08	383.17	372.50

**Table 6:** DSC analysis of the second heating cycle for the composite in pellet format: with and without thermomechanical processing; Tg: glass transition temperature; Tc-1 and Tc-2: crystallization temperature; Tpm: peak melting temperature.

		Tg		Tc-1		Tc-2		Tpm	
Temperatures (°C): 2nd Heating		Onset	Endset	Onset	Peak	Onset	Peak	Onset	Peak
	Neat PLA	57.71	60.23	92.16	96.43	153.9	156.57	166.13	172.48
(1) WOTP	PLA-5% WOTP-2100	57.88	60.09	93.17	97.41	154.02	156.71	165.97	172.34
	PLA-10% WOTP-2100	58.24	61.71	87.96	96.08	153.07	158.16	166.52	175.74
	PLA-15% WOTP-2100	57.93	61.07	91.83	98.3	153.71	157.86	166.04	174.94
(2) WTP		Tg		Tc-1		Tc-2		Tpm	
		Onset	Endset	Onset	Peak	Onset	Peak	Onset	Peak
	PLA-5% WTP-2100	57.26	59.46	92.96	98.69	153.33	156.58	166.05	172.21
	PLA-10% WTP-2100	57.13	59.82	90.95	99.45	153.09	156.65	166.03	172.4
	PLA-15% WTP-2100	57.00	60.13	84.49	96.95	151.89	156.39	165.6	172.59
(3) WTP		Tg		Tc-1		Tc-2		Tpm	
		Onset	Endset	Onset	Peak	Onset	Peak	Onset	Peak
	PLA-5% WTP-800	56.97	59.85	91.46	97.99	152.78	156.22	165.78	172.18
	PLA-10% WTP-800	56.73	60.13	84.48	96.28	151.99	156.22	165.53	172.38
	PLA-15% WTP-800	56.53	59.89	88.74	96.29	152.57	156.54	165.63	172.17

## Data Availability

The original contributions presented in this study are included in the article. Further inquiries can be directed to the corresponding authors.

## Abbreviations

The following abbreviations are used in this manuscript:

PLA	Polylactic acid
MEX	Material extrusion
TGA	Thermogravimetric analysis
DSC	Differential scanning calorimetry
SEM	Scanning electron microscope
AM	Additive manufacturing
FDM	Fused deposition modeling
TP	Thermomechanical processing
WTP	With thermomechanical processing
WOTP	Without thermomechanical processing
